# Do You Mind? Examining the Impact of Psychoeducation Specificity on Perceptions of Mindfulness-Based Programs

**DOI:** 10.3390/ijerph19159621

**Published:** 2022-08-04

**Authors:** Nicole Del Rosario, Shadi Beshai

**Affiliations:** 1 Department of Psychology, University of Manitoba, Winnipeg, MB R3T 2N2, Canada; 2 Department of Psychology, University of Regina, Regina, SK S4S 0A2, Canada

**Keywords:** mindfulness-based program, perceptions, acceptability, credibility, psychoeducation

## Abstract

Objective: Mindfulness-based programs (MBPs) cultivate the capacity for mindfulness, defined as nonjudgmental acceptance and awareness of present-moment experience. Mindfulness has been associated with a host of benefits for users, such as improved indices of mental well-being. We examined public perceptions of acceptability (i.e., how appropriate the treatment is for a given problem) and credibility (i.e., how logical and convincing a treatment seems) of MBPs as a form of mental health intervention. The main objective of this study was to examine whether higher specificity of psychoeducational content improved perceptions of the acceptability and credibility of MBPs. Methods: Participants (*n* = 188; female% = 39.4) were recruited online and randomly assigned to one of two conditions. In one condition, participants received balanced and evidence-based psychoeducation specific to MBPs for mental health. In the other condition, participants received general information about psychological treatments for mental health. Acceptability and credibility perceptions were measured by questionnaires across time (pre-and post-psychoeducation) and across specificity conditions (specific vs. general psychoeducation). Results: Participants randomized to the general, but not the specific, psychoeducation-endorsed higher scores of acceptability of MBPs post-psychoeducation. Further, participants endorsed higher scores of MBP credibility post-psychoeducation, regardless of the specificity of psychoeducation provided. Conclusions: Perceptions of the acceptability of MBPs were improved following exposure to general psychoeducation, and perceptions of the credibility of MBPs were improved following psychoeducation, regardless of specificity. Examining public perceptions of MBPs is important for informing strategies to support access to and use of MBPs.

## 1. Introduction

There has been an increased interest in the clinical applications of mindfulness [1] by way of mindfulness-based programs (MBPs). Mindfulness has been defined as paying attention to present-moment experiences with purpose, openness, and non-judgement [2]. Mindfulness techniques (e.g., focus on breath or body) have been incorporated within health settings in the form of MBPs to help individuals respond more skillfully to their experiences [3]. Benefits of mindfulness practice include increased well-being [4] and psychological resilience [5]. Despite these benefits, less is known about whether individuals are interested in engaging in MBPs as a form of psychological treatment.

MBPs share core principles guiding their development [6]: (1) A foundation informed by theories and practices that span from contemplative traditions, science, and major disciplines (e.g., medicine, psychology); (2) a model of human experience which addresses the causes of human distress and pathways to relieving distress; (3) intention to develop a new relationship with experience characterized by decentering and present moment focus; (4) support the development of greater self-regulation and qualities like compassion; and (5) the requirement of a regular mindfulness meditation practice which may include a focus on breath or body. Mindfulness-based stress reduction (MBSR) and mindfulness-based cognitive therapy (MBCT) are prominent first-generation MBPs and are only two examples of the diverse forms MBPs can take. In addition to the state conceptualization of mindfulness noted above, mindfulness may also be conceptualized as a trait with individuals embodying varying capacities to be mindful [7]. The diversity of MBPs and the inconsistent use of the term “mindfulness” may increase confusion and proliferating misinformation among general consumers seeking mental health interventions [8]. Providing members of the general public with access to evidence-based information specific to MBPs may coincide with favorable perceptions of MBPs as a treatment and may potentially lead to greater enthusiasm for and uptake of such programs.

### 1.1. Treatment Perceptions

Many factors may influence individual willingness to engage in a MBP, even when such interventions are accessible. Researchers have linked perceptions of psychological treatments to their therapeutic outcomes. For example, accommodating client preference in psychotherapy has been associated with fewer treatment dropouts and more positive treatment outcomes [9]. An individual may not adhere to or complete treatment protocols perceived as unacceptable, underscoring the need to investigate treatment preferences among clients [10]. Patient treatment expectations may develop from perceptions of treatment credibility [11], making credibility assessments a potentially significant part of patient outcome expectations and, consequently, outcomes themselves. Treatment perceptions, such as those of acceptability and credibility, have been identified as relevant attitudinal barriers to treatment engagement [12]. Treatment acceptability refers to a consumer’s perception of whether a treatment is appropriate for a given problem; specifically, if the treatment appears fair, reasonable, non-intrusive, and if it meets the conventional notions of what a treatment should be [13,14]. On the other hand, treatment credibility is defined as how plausible, logical, and convincing a treatment seems to a potential participant [15,16]. Accordingly, there is a need to investigate perceptions of MBPs and develop brief but effective strategies to maximize accurate and favorable perceptions of MBPs.

While research on the application of MBPs has been present for the past few decades, the popularization of MBPs is ever expanding [17]. Mindfulness-based interventions have been described as an “emerging phenomenon” [18]. The relatively new emergence of MBPs may coincide with insufficient public knowledge. Understanding public perceptions of emergent psychological therapies may be informed by examining a similar line of research among other developing interventions. For example, Internet-delivered cognitive behavior therapy (ICBT) is a relatively novel psychological therapy option with relatively low uptake [19]; nevertheless, educational materials appear effective for increasing ICBT acceptance and outcome expectations [19,20,21]. The current study was designed to investigate whether exposure to brief psychoeducational material impacts perceptions of the acceptability and credibility of MBPs.

We also examined whether presenting people with specific information about MBPs can improve perceptions of these programs more so than the provision of general information regarding mental health programs. Questions arise regarding what information may be beneficial for the general public when potentially considering engaging in an MBP. Schofield et al. examined lay perceptions of the term “evidence-based” and found that only half of the sample accurately identified the meaning of the term [22]. Researchers suggested the need for future research to examine content presented in psychoeducational material. One area that remains unclear is the impact of content specificity on perceptions of treatment. Jorm et al. evidenced the effectiveness of specific rather than general information in improving attitudes toward treatment [23]. Participants in the study were presented with either an evidence-based consumer guide to treatments for depression or a general brochure on depression. There were significant differences between groups regarding changes in beliefs about the effectiveness of certain interventions (i.e., CBT, electroconvulsive therapy, St John’s wort) before and after participants were provided with informational material in which specific information was related to attitudes of increased effectiveness. More recently, Mitchell and Gordon found that university students’ perceptions of CBT improved following a 30-min video demonstration delivered via a CD-ROM [24]. Beshai et al. have also demonstrated that participants’ views of CBT credibility improved following exposure to brief psychoeducational materials specific to CBT [25].

### 1.2. Current Study

While there has been increased interest in the clinical applications of mindfulness [1] and evidence of mindfulness as effective for improving mental health [26], the nature and level of interest among members of the general public in MBPs as psychological treatment remain unknown. Public promotion of evidence-based mental health interventions is a concerted effort within the mental health literacy movement. Accordingly, treatment dissemination efforts would be supported by providing members of the public with psychoeducation about the nature and efficacy of MBPs [27].

In the current study, we hoped to address the aforementioned gaps in the literature concerning perceptions of MBPs. Specifically, the objective of the current study was to: (a) Explore whether perceptions of the acceptability and credibility of MBPs improve when participants are presented with balanced and evidence-based psychoeducation; and, (b) whether improvement is dependent on the specificity of information presented. Accordingly, acceptability and credibility perceptions were measured across time (pre-and post-psychoeducation) and across specificity conditions (specific vs. general psychoeducation). First, we hypothesized an interaction effect such that participants exposed to MBP-specific psychoeducation are predicted to report significantly improved perceptions of MBPs (credibility and acceptability) post-psychoeducation compared to those exposed to general psychoeducation. Second, we hypothesized a main effect of time, whereby perceptions of MBPs (acceptability and credibility) will improve following exposure to educational materials in both groups (general vs. specific).

## 2. Method

### 2.1. Participants

Participants were recruited in February 2020 through TurkPrime [28], which is a versatile extension of Amazon’s Mechanical Turk (MTurk), a crowdsourcing online platform [29]. There is evidence that crowdsourced convenience samples are more representative of the general population than university student samples [30]. Such online platforms are now commonly used in clinical psychological research [31]. Eligible participants were required to reside in an English-speaking country (i.e., Canada, United States, United Kingdom, New Zealand, and Australia), speak English proficiently, and be 18 years of age or older. A total of 251 participants provided consent and completed study questionnaires; however, several participants (*n* = 58) were excluded from analyses for failing to pass the attention checks and for missing 20% of items or greater on average on each measure.

A total of 188 participants were included in the final analyses. Participants ranged in age from 19 to 69, with a *M*_age_ = 38.2 (*SD* = 11.20). The majority of participants identified as male (*n* = 114; 60.6%), white (*n* = 146; 77.7%), and resided in the United States of America (*n* = 183; 97.3%).

### 2.2. Procedure

The University of Regina’s Research Ethics Board approved the current study (REB File #:2019-198). After providing consent and completing the demographic information form, participants were presented with self-report questionnaires of treatment acceptability and credibility in randomized order. Participants were then randomly assigned (using Qualtrics randomization algorithms) to be presented with either (1) psychoeducation in the form of a written description of MBPs, including a list of the programs’ advantages and disadvantages as a mental health intervention; or (2) psychoeducation regarding psychotherapy not specific to MBPs in the same format (i.e., a general psychoeducation intervention). All participants were then asked to complete treatment acceptability and credibility questionnaires a second time, also in randomized order. After completing the questionnaires, participants were thanked for participating, debriefed, and compensated for their participation.

### 2.3. Materials

#### 2.3.1. The Modified Treatment Acceptability/Adherence Scale (TAAS) 

The TAAS is an 8-item, self-report measure of treatment acceptability [14]. Statements (e.g., “If I began this treatment, I would be able to complete it”) are rated on an 8-point Likert scale, from “1” or “Disagree strongly” to “8” or “Agree strongly” with total summed scores ranging from 8 to 56. Four of the items are reversed scored, such that greater scores are indicative of greater treatment acceptability. For the current study, the word “treatment” in all items was replaced with “mindfulness-based programs”. Furthermore, as the scale was originally developed to assess anxiety treatment acceptability, specific wording referencing “fear/anxiety” in items 6 and 7 was changed to refer to “mental health” and treatment-specific items (“I would prefer to try another type of psychological treatment instead of this one” and “I would prefer to receive medication from my fear/anxiety instead of this treatment”) were not included. The TAAS has demonstrated good internal consistency in both undergraduate [14] and clinical samples [32] with Cronbach alphas of 78 and 88 respectively. Beshai et al. report high internal consistency for a similarly modified TAAS used to assess depression treatments in a crowdsourced convenience sample with Cronbach alphas ranging from 86 to 90 pre-psychoeducation and 93 to 95 post-psychoeducation across the two studies [25]. In the current sample, Cronbach alphas for the TAAS were 89 pre-psychoeducation and 92 post-psychoeducation.

#### 2.3.2. The Modified Credibility/Expectancy Questionnaire (CEQ)

The CEQ is a 6-item, self-report measure of an individual’s beliefs of how logical a treatment is (credibility) and beliefs of its expected efficacy (expectancy) [16]. The credibility subscale is based on three items using the 9-point scale (from “1” or “Not at all logical” to “9” or “Very logical”) which measure how logical the therapy seems, how successful it will be in reducing symptoms, and how confident one would be in recommending it to a friend with similar symptoms. Total scores for the credibility factor range from 3 to 27. The two-factor model of the CEQ has been supported by factor analysis [16]. Good internal consistency of items within each factor have been reported in a clinically anxious sample, α = 0.81 for credibility; α = 0.79 for expectancy [16], and with an undergraduate sample, α = 0.67 for credibility; α = 0.92 for expectancy [14]. For the current study, “treatment” in all items was replaced with “mindfulness-based programs”. Beshai et al. reported high internal consistency of both factors of a modified CEQ for depression treatments in a crowdsourced convenience sample, similar to current study participants with Cronbach’s alpha ranging from 0.95 to 0.96 pre-psychoeducation and 0.86 to 0.91 post-psychoeducation across the two studies [25]. In the current study, Cronbach alphas for the CEQ-credibility subscale were 0.90 pre-psychoeducation and 0.93 post-psychoeducation.

### 2.4. Psychoeducation

Consistent with previous research [12,20,25], feedback regarding the psychoeducational materials was solicited from four psychologists who were experts in MBPs (*n* = 2) or psychotherapy generally (*n* = 2). The experts were asked to read the psychoeducation materials and provide general feedback and quantitative ratings about the material clarity, accuracy, and comprehensiveness. The feedback was used to revise the psychoeducational materials.

#### 2.4.1. MBP Specific Psychoeducation

The MBP psychoeducational material was designed to provide balanced, evidence-based, and specific information about MBPs. The psychoeducation provided participants with a 472-word written description that described defining features of MBPs, how MBPs are thought to enhance mental health, and the nature and typical course of an MBP. Following the description of MBPs, an evidence-based list of five advantages (e.g., “*According to several scientific studies, mindfulness training has been linked to benefits for anxiety and depression symptoms*”) and five disadvantages (e.g., “*While some participants report relaxation following mindfulness training exercises, it is not uncommon for participants to report unpleasant reactions like agitation, anxiety, and discomfort*”) of MBPs were presented. The MBP psychoeducation description is based on the framework provided by Crane et al. [6]. Information specific to MBSR was taken from Kabat-Zinn [2]. Advantages and disadvantages were consulted from theoretical and research sources [4,26,33,34]. 

#### 2.4.2. General Psychoeducation

The General Psychoeducation Intervention material was designed to create a comparison group to test the generic impact of providing psychoeducation. The General Psychoeducation Intervention material provided participants with a 373-word written description that described effects of receiving psychotherapy for mental health, with a one sentence description of typical MBP time commitment and session length alongside evidence-based lists of five advantages (e.g., “*Psychotherapy can teach coping skills that are applicable for future life stressors*”) and five disadvantages (e.g., “*To effectively treat certain conditions, such as anxiety disorders, patients may experience temporary surges in uncomfortable emotions, such as anxiety*”) of psychotherapy in general. The General Psychoeducation Intervention material is based on the American Psychological Association and the American Psychiatric Association webpages intended for public use [35,36]. 

### 2.5. Data Analyses

Prior to conducting the statistical analyses, the data were checked for missing values, outliers, and evidence of violations of normality in the data. Data were inspected for missing values by visually inspecting the data set as well as checking the sample size for each variable of interest. Missing data were less than 1%. Mean person imputation was used to calculate mean summed scores for participants with less than 20% of missing responses to a scale. Skewness and kurtosis for each scale were examined using the recommended cut-offs to gauge the extent to which the data deviated from normality (recommended cut-off of ±1 for skewness and ±1.5 for kurtosis) [37]. None of the variables in question had skewness and kurtosis values that exceeded the recommended cut-offs. A visual inspection of histograms also supported assumptions of data normality.

Independent sample *t*-tests were also used to ensure scores of acceptability and credibility did not significantly differ at pre-psychoeducation. The first and second hypotheses were assessed with two mixed-factor analyses of variance (ANOVAs) each for acceptability and credibility assessing for changes in perceptions of MBPs over time and across conditions. The between-subject factor was intervention condition (i.e., MBP-specific psychoeducation or general psychoeducation) and the within-subject factor was time (i.e., pre- and post-psychoeducation). To control for potentially confounding retesting effects, scores across time (i.e., pre- and post-psychoeducation) were compared between groups to account for retesting effects of the TAAS and CEQ scales. Within condition paired sample *t*-tests were used to further assess statistically significant interactions and main effects. Alpha levels were set at 0.05, and effect size estimates were calculated where appropriate.

## 3. Results

Sample characteristics are displayed in Table 1. Descriptive statistics of measures are displayed in Table 2.

### 3.1. Differences in Pre-Psychoeducation Perceptions of Acceptability and Credibility

An independent samples *t*-test evidenced no statistically significant difference on pre-psychoeducation scores of acceptability between participants assigned to the MBPs-specific (*M* = 40.25, *SD* = 7.81) or general psychoeducation condition (*M* = 39.02, *SD* = 10.70); t(186) = −0.90, p = 0.368, d = 0.132. Likewise, pre-psychoeducation scores on the measure of credibility did not differ among participants in the specific (*M* = 16.88, *SD* = 5.66) or general psychoeducation condition (*M* = 17.70, *SD* = 5.68); t(186) = −0.99, *p* = 0.323, d = 0.143.

### 3.2. Effects of Psychoeducation on MBP Acceptability/Adherence Scores

**Figure 1:**
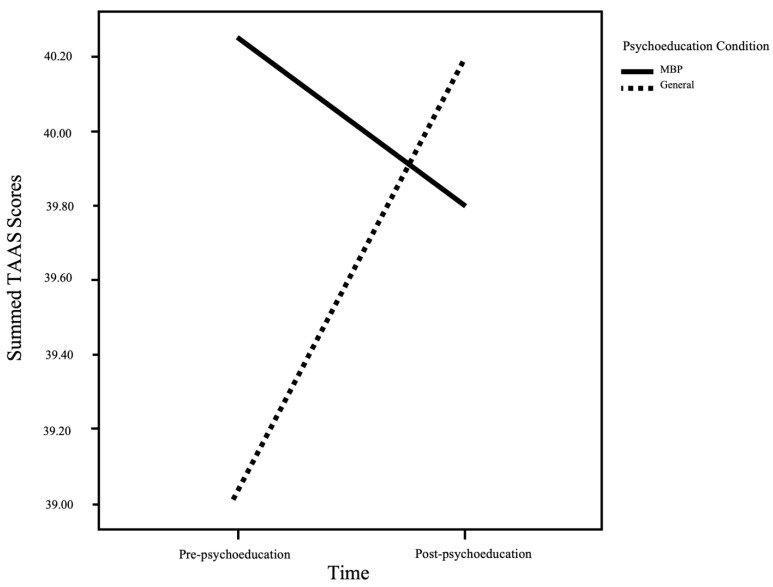
MBP acceptability summed scores pre-psychoeducation and post-psychoeducation, stratified by psychoeducation condition (specific vs. general). *Note*. MBP = mindfulness-based programs; TAAS = Treatment Adherence and Acceptance Scale.

A mixed ANOVA revealed a significant interaction between psychoeducation condition and time on TAAS scores (see Figure 1). Paired samples *t*-tests evidenced participants in the general psychoeducational condition endorsed statistically significantly higher TAAS scores post-psychoeducation (*M* = 40.18, *SD* = 11.37) relative to pre-psychoeducation (*M* = 39.02, *SD* = 10.70); *t*(91) = −3.07, *p* = 0.003, *d* = 0.321. Whereas participants presented with the MBP-specific psychoeducation did not evidence statistically significant changes in their perceptions of MBPs acceptability from pre- (*M* = 40.25, *SD* = 7.81) to post-psychoeducation (*M* = 39.80, *SD* = 10.46); *t*(95) = 0.75, *p* = 0.453, *d* = 0.077. Mixed ANOVA results are displayed in Table 3. The analysis revealed no statistically significant main effect of time, or psychoeducation condition, on TAAS scores.

### 3.3. Effects of Psychoeducation on MBP Credibility Scores

**Figure 2:**
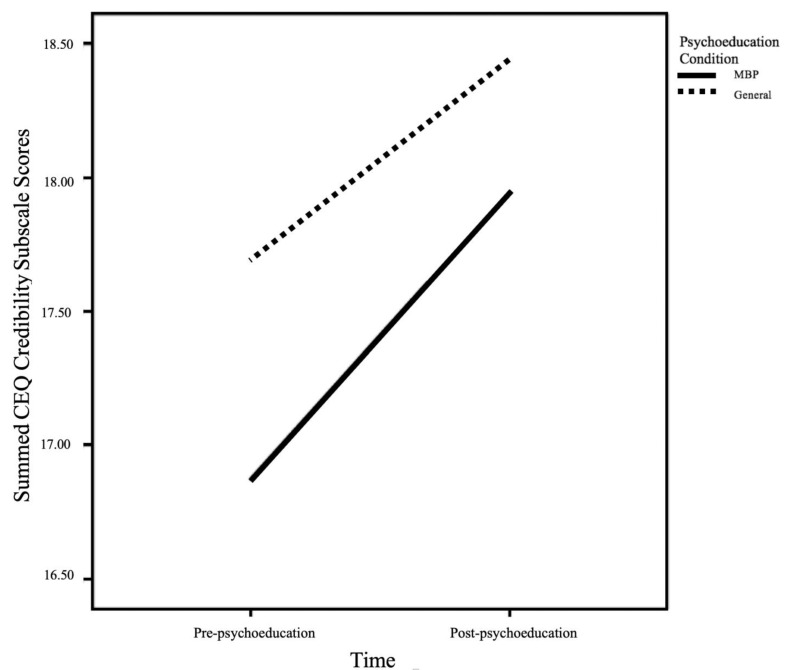
MBP credibility summed scores pre-psychoeducation and post-psychoeducation, stratified by psychoeducation condition (specific vs. general). Note. MBP = mindfulness-based programs; CEQ = Credibility and Expectancy Questionnaire.

The second mixed ANOVA used summed CEQ Credibility scores as the dependent variable. The interaction between psychoeducation condition and time was not statistically significant, (see Figure 2). Mixed ANOVA results are displayed in Table 3. There was also no statistically significant main effect for psychoeducation condition. However, there was a statistically significant main effect of time, such that post-psychoeducation scores (*M* = 18.19, *SD* = 5.75) were significantly higher than pre-psychoeducation scores (*M* = 17.28, *SD* = 5.67) across the psychoeducation conditions.

## 4. Discussion

There is a growing body of evidence supporting the effectiveness of MBPs as an intervention for various mental health concerns [34,38,39]. Despite the effectiveness of MBPs, several factors may influence participant interest in engaging with such programs. Perceptions of the acceptability and credibility of MBPs as a mental health intervention are important factors influencing treatment engagement and outcomes. For example, individuals who perceive a protocol as unacceptable may choose not to begin this protocol or discontinue it prematurely [10]. Positive perceptions of credibility are associated with positive treatment expectations and, subsequently, better clinical outcomes for program completers [15]. As such, investigating perceptions of MBPs may be critical for supporting treatment uptake and engagement. Knowledge of treatment perceptions can inform the development of effective strategies to maximize positive perceptions for treatment promotion and dissemination. Given the emerging popularity of MBPs, the definitions of mindfulness and MBPs are evolving. As such, we examined the impact of general and specific psychoeducational material on perceptions of MBPs.

### Brief Psychoeducation for Perceptions of the Acceptability and Credibility of MBPs

To assess the impact of providing brief MBP-specific psychoeducation on public perceptions of MBPs, we administered measures of treatment acceptability and credibility before and after participants were presented with either general or MBP-specific psychoeducation. There were no statistically significant differences in the initial perceptions of acceptability and credibility of MBPs across groups, indicating successful randomization.

The obtained results were not supportive of our first main hypothesis. Despite the significant interaction effect of time and specificity condition on TAAS scores, participants randomized to the general condition, but not MBP-specific condition, reported significantly improved acceptability scores after receiving the psychoeducation. General psychoeducation was found to be more effective in increasing perceptions of MBPs’ acceptability than specific psychoeducation, which aligns with previous findings from Beshai et al. [25]; these researchers found that perceptions of the acceptability of CBT decreased as a function of CBT-specific psychoeducation. However, the current results were inconsistent with previous evidence that providing specific psychoeducation material on mental health treatments enhances treatment perceptions [23]. 

It is possible that an understanding of the specific foundational components of MBPs may be unappealing or unnecessary to improve perceptions among general consumers. It may also suggest that academic critiques regarding whether to include an explicit connection to Buddhist practices in MBPs are not a focal concern to a consumer in the general public. Improved perceptions of the acceptability of MBPs among participants assigned to the general psychoeducation group may indicate that these perceptions are malleable even after exposure to brief psychoeducation; however, the results contrast previous evidence that specifics may be more influential [23]. It is plausible, given our results, that simply presenting MBPs as a possible mental health intervention amongst evidence-based psychotherapy information positions MBPs in a way that meets the conventional notions of mental health treatment and thus appears acceptable for potential consumers.

The study results partially supported our second hypothesis that perceptions of MBP acceptability and credibility would improve across time regardless of specificity condition. Specifically, we found perceptions of MBPs credibility improved following exposure to psychoeducational materials in both groups (general vs. specific), whereas there was no main effect of time on acceptability scores. Accordingly, we found that providing brief written psychoeducation, regardless of content specificity, produced statistically significant improvements in the perceived credibility of MBPs for mental health. In this way, credibility may be more malleable than acceptability as improvement was not associated with specificity, which is consistent with previous research on CBT [25]. Potential participants may only require MBPs to be cited alongside other evidence-based mental health interventions to shift credibility perceptions.

Overall, our findings suggest preliminary support for providing general psychoeducation to improve perceptions of the acceptability and credibility of MBPs. The general psychoeducation condition evidenced improvement in perceptions of both acceptability and credibility of MBPs. Providing specific education was not more effective for improving perceptions than general psychoeducation for credibility and in fact, was less effective than general psychoeducation for improving perceptions of acceptability. These results suggest that specific psychoeducation may not be appropriate for enhancing perceptions of acceptability among lay consumers. It is important to note that while statistically significant, effect sizes noted in Table 3 are small which may suggest the limited practical significance of a single-session intervention in improving perceptions of MBPs.

Our findings also support Corrigan’s described limitations of using educational approaches to improve mental health literacy [40]. Health literacy is “the degree to which individuals can obtain, process, understand, and communicate about health-related information needed to make informed health decisions” [41]. Mental health literacy emerged from the larger field of health literacy and describes knowledge and beliefs that can improve recognition, management, or prevention of mental disorders [42]. Researchers reported a specific need for mental health literacy as this type of community knowledge significantly lags behind physical health literacy [43]. Encouragingly, extant evidence suggests interventions can be successful in increasing mental health literacy [44]. Increasing mental health literacy among the general public is essential for supporting consumer decision-making [45].

Corrigan critiqued mental health literacy educational campaigns, suggesting that a “more is better” approach to education, in that more information is believed to inherently be better, may overwhelm the public [40]. Indeed, researchers support this concern by suggesting that individuals with limited health literacy may experience cognitive overload when trying to read and process health messages [46]. The current results also suggest against a “more is better” approach, indicating that providing general information can be sufficient for changing public perceptions of the acceptability and credibility of MBPs. Our findings also support previous results from Meppelink et al in which researchers found that simple health materials designed for individuals with low health literacy were effective for increasing health literacy in both low and high heath literacy audiences [47].

The current study is, to our knowledge, the first to explore public perceptions of MBPs and has several implications regarding these perceptions and MBP dissemination. Previous research indicates that perceptions of treatment acceptability and credibility are key components that impact engagement with a therapy [12]. By investigating baseline acceptability and credibility rates of MBPs, our findings provide important insights regarding brief psychoeducation strategies for MBP promotion and dissemination. Public promotion of MBPs supports treatment seekers for clinical interventions. In a broader sense, public promotion of MBPs also supports mental health literacy efforts for the general consumer, even among those individuals that may not be currently seeking mental health programs. An understanding of the public’s perceptions of MBPs also shapes policy decisions, which consequently impacts funding and program access. Negative perceptions of MBPs are potential barriers to treatment engagement that should be considered when examining strategies to enhance perceptions of MBPs as a psychological treatment [9].

## 5. Limitations and Future Directions

While the current study has a number of strengths, our findings should be considered in light of limitations which provide opportunities for future research in this field. First, the single-session nature of our design does not allow for inferences into the potential long-term impacts of psychoeducation. Future studies should attempt to establish causality using longitudinal designs across data collection sessions. Second, the sample was convenient in nature. Crowdsourced convenience samples appear more representative of the general population than university student samples but still tend to be younger, less religious, and more liberal than the general population [30], which also reflects differences between Internet and non-Internet users [48]. Use of a crowdsourced convenience sample may limit the generalizability of findings to clinical or other underrepresented populations. Despite the limitations of this type of sample, the current study was designed to assess general public perceptions; as such, it is appropriate for the current investigation. Future research would benefit from examining how these perceptions may differ for individuals actively seeking mental health intervention. Third, the current sample consisted mainly of white males, potentially limiting the generalizability of the results to more diverse populations. Future research should replicate the current study’s results with samples more representative of general population parameters. Fourth, the study measures assess treatment *perceptions* rather than help-seeking *intentions* and help-seeking *behaviors*. It is important that researchers examine whether perception-related variables predict actual engagement behavior in MBPs for mental health rather than just perceptions toward such programs. Subsequent research should pair assessments of MBP perceptions with actual engagement and completion of an MBP. This research may also address the question of what statistical change in score for a measure of perception is related to change in behavior. Further, quantitative assessment of preference for MBPs versus other treatment modalities may enhance protocols for identifying (a) individuals who may be interested in a referral to this type of intervention; and (b) key factors individuals find appealing that may be presented in psychoeducational materials.

Despite these limitations, the current study contributes considerably to this growing literature for several reasons. This study is the first of its kind to directly examine public perceptions of MBPs and whether these perceptions can be modified with brief psychoeducational material.

## 6. Conclusions

This research added meaningfully to the literature on mindfulness by examining public perceptions of MBPs and whether brief psychoeducational materials can shift these perceptions. Our findings indicate that perceptions of credibility improve across time, regardless of the specificity of the psychoeducation presented, and that perceptions of acceptability improved following the presentation of general psychoeducation. Participants exposed to MBP-specific psychoeducation did not evidence significantly greater improvements in MBP perceptions, contrary to previous research by Jorm et al. [23]. In summary, this study provided support that perceptions of the acceptability and credibility of MBPs are malleable to change following exposure to general psychoeducational material. Although perceptions of acceptability and credibility have been identified as important factors in treatment engagement [12], our findings suggest that these two factors respond differently to specific psychoeducational material and possibly, general psychoeducation may be more effective than more intensive interventions. In addition to generating new knowledge about the influence of psychoeducation on shifting perceptions of acceptability and credibility of MBPs, these results could facilitate improvement in how MBPs are disseminated and promoted among public and clinical audiences. Future research, guided by the results of this study, may begin designing relevant psychoeducational campaigns for enhancing perceptions of acceptability and credibility of MBPs.

## Figures and Tables

**Table 1:** Demographic characteristics of the participant sample.

Psychoeducation Condition	Total Sample *(n* = 188)		*General* *(n =* 92)	*Specific* *(n =* 96)
**Age**	*M*	*SD*	*M*	*M*
	38.21	11.17	38.85	37.59
**Gender**	*n*	%	*n*	*n*
Female	74	39.4	36	38
Male	114	60.6	56	58
**Ethnicity**				
African	13	6.9	6	7
Asian	16	8.6	5	11
Hispanic	11	5.9	5	6
White	146	77.7	76	70
Other	2	1.1	0	2
**Marital Status**				
Single	88	46.8	37	51
Cohabitating	20	10.6	14	6
Dating	14	7.4	6	8
Married	54	28.7	32	22
Separated/Divorced/Widowed	12	6.4	3	9
**Country of Residence**				
Canada	4	2.1	2	2
United States of America	183	97.3	89	94
United Kingdom	1	0.5	1	0
**Current Employment Status**				
Employed Full-time	143	76.1	74	69
Retired Full-time	3	1.6	2	1
Student	4	2.1	1	3
Employed part-time	21	11.2	8	13
Homemaker	4	2.1	2	2
Unemployed	11	5.9	5	6
Long-term disability	2	1.1	0	2
**Highest Level of Education**				
High school diploma	54	28.7	26	28
College certificate or diploma	30	16.0	20	10
University undergraduate degree(s)	77	41.0	37	40
University professional or graduate degree(s)	27	14.4	9	18

*Note*. Some subcategories statistics were not included in this table.

**Table 2:** Descriptive statistics of measures.

	Pre-Psychoeducation		Post-Psychoeducation	
Measures	*M (SD)*		*M (SD)*	
Psychoeducation Condition	*General*	*Specific*	*General*	*Specific*
TAAS	39.02 (10.70)	40.23 (7.81)	40.18 (11.37)	39.80 (10.46)
CEQ Credibility	17.70 (5.68)	16.88 (5.66)	18.45 (6.06)	17.95 (5.45)

*Note*. TAAS = Treatment Adherence and Acceptance Scale; CEQ = Credibility and Expectancy Questionnaire.

**Table 3:** Mixed ANOVA results for TAAS and CEQ scores over time (pre-post psychoeducation) and across conditions (MBP-specific psychoeducation or general psychoeducation).

	F-Statistic	*p*	*η_p_^2^*
**TAAS**			
Effect of Time (Pre vs. Post)	1.01	0.316	0.005
Effect of Group (MBP specific vs. General)	0.09	0.769	0.000
Time X Group	5.12	0.025 *	0.030
**CEQ Credibility**			
Effect of Time (Pre vs. Post)	11.23	0.001 **	0.057
Effect of Group (MBP specific vs General)	0.70	0.404	0.004
Time X Group	0.35	0.553	0.002

*Note*. MBP = mindfulness-based programs; TAAS = Treatment Adherence and Acceptance Scale; CEQ = Credibility and Expectancy Questionnaire * = significant at 0.05 alpha level ** = significant at the 0.01 alpha level.

## Data Availability

The data presented in this study are available on request from the corresponding author.
